# Characterisation and culture of primary human nasal epithelial cells and the influence of Interferon-gamma

**DOI:** 10.1186/2045-7022-4-S2-P21

**Published:** 2014-03-17

**Authors:** Eva Waltl, Julia Eckl-Dorna, Regina Selb, Rudolf Valenta, Verena Niederberger

**Affiliations:** 1 Medical University of Vienna, Department of Otorhinolaryngology, Vienna, Austria; 2 Medical University of Vienna, Department of Pathophysiology and Allergy research, Vienna, Austria

## Background

The nasal epithelium with its tight junctions (TJs) represents an important barrier against the penetration of exogenous substances (e.g. allergens, pollutants and pathogens). Damage of the epithelium allows higher amounts of inhaled allergens and pollutants to penetrate the mucosa. Our laboratory has previously used a bronchial epithelial cell line to investigate epithelial damage by various factors [cigarette smoke (Gangl et al., 2009) and cytokines (Reisinger et al., 2005)]. Cultures of primary epithelial cells obtained from nasal epithelium should be superior in resembling the natural situation in the nose and allow a comparison of epithelia from non-allergic and allergic patients. Our aim was to establish primary human nasal epithelial cell culture in a transwell system and to investigate the epithelial barrier function and the properties of the nasal epithelium. Additionally we compared different epithelial cell culture systems to investigate damage and repair in primary human nasal epithelial cells.

## Methods

We obtained nasal tissue samples during routine surgeries and cultured primary human nasal epithelial cells in a transwell system with air-liquid interface as a surrogate of an intact respiratory epithelium. In order to investigate epithelial damage we used the TH1-derived cytokine interferon-γ (IFN-γ), which has previously been shown to impair the barrier function of the nasal mucosa. Scratch testing was set up as a standard method to investigate the effect of physical damage on cell monolayers. The phenotype of the cells was confirmed by light microscopy, flow cytometry and fluorescence staining of cells cultured on transwell membranes.

## Results

Primary human nasal epithelial cells gained confluence and displayed high TER after approximately 8 days of incubation in transwell inserts. Incubation of cells with IFN-γ led to a reduction of TER within 24 hours of incubation. In scratch tests, the untreated cell monolayers gained confluence at the latest after 96 hours hours after performing damage. Repair of cell layers was inhibited by IFN-γ treatment.

## Conclusion

We established cell cultures based on primary human nasal epithelial cells and reproducible techniques to investigate the epithelial barrier function. Supported by projects of the Austrian Science Fund (FWF, P4613, P4605) and the DK program MCCA.

